# The Worldwide Association between Television Viewing and Obesity in Children and Adolescents: Cross Sectional Study

**DOI:** 10.1371/journal.pone.0074263

**Published:** 2013-09-25

**Authors:** Irene Braithwaite, Alistair W. Stewart, Robert J. Hancox, Richard Beasley, Rinki Murphy, Edwin A. Mitchell, the ISAAC Phase Three Study Group

**Affiliations:** 1 Medical Research Institute of New Zealand, Newtown, Wellington, New Zealand; 2 School of Population Health, The University of Auckland, Auckland, New Zealand; 3 Department of Preventive & Social Medicine, Dunedin School of Medicine, University of Otago, Dunedin, New Zealand; 4 Department of Medicine, Faculty of Medicine and Health Sciences, The University of Auckland, Auckland, New Zealand; 5 Department of Paediatrics: Child and Youth Health, Faculty of Medicine and Health Sciences, The University of Auckland, Auckland, New Zealand; University of Missouri-Kansas City, United States of America

## Abstract

**Background:**

Studies exploring the effect of television viewing on obesity throughout childhood are conflicting. Most studies have been confined to single high-income countries. Our aim was to examine the association between television viewing habits and Body Mass Index (BMI) in adolescents and children in a multicentre worldwide sample.

**Methods:**

In the International Study of Asthma and Allergies in Children Phase Three, adolescents aged between 12 and 15 years completed questionnaires which included questions on television viewing habits, height and weight. Parents/guardians of children aged between 5 and 8 years completed the same questionnaire on behalf of their children. The questionnaire asked “During a normal week, how many hours a day (24 hours) do you (does your child) watch television?” Responses were categorised as; “short” (<1 hour), “moderate” (1 to ≤3 hours), “long” (3 to ≤5 hours) and “prolonged” (>5 hours).

**Findings:**

207,672 adolescents from 37 countries and 77,003 children from 18 countries provided data. Daily television viewing in excess of one hour was reported in 89% of adolescents and 79% of children. Compared with adolescents in the short viewing group, those in the moderate, long and prolonged groups had BMIs that were 0.14 kg/m^2^, 0.21 kg/m^2^, 0.30 kg/m^2^ and 0.08 kg/m^2^, 0.16 kg/m^2^ and 0.17 kg/m^2^ larger for females and males respectively (both P<0.001). Compared with children in the short viewing group, those in the moderate, long and prolonged groups had BMIs that were 0.24 kg/m^2^, 0.34 kg/m^2^, 0.36 kg/m^2^ and 0.19 kg/m^2^, 0.32 kg/m^2^ and 0.36 kg/m^2^ larger for females and males respectively (both P<0.001).

**Interpretation:**

Increased television viewing hours were positively associated with BMI in both adolescents and children with an apparent dose response effect. These findings extend the evidence that television viewing contributes to increased BMI in childhood.

## Introduction

There is increasing concern about the rising prevalence of obesity worldwide and its health implications [1] [2] [3]. The rising prevalence of childhood obesity is marked [4] [5] [6] and there are now well documented concerns about the future health implications of obesity in childhood [7] [8] [9]. This problem has been identified in low and middle income countries as well as affluent countries [3] [6] [10] [11].

While potential contributors to the problem of childhood obesity are multiple and complex, increasing television viewing in children has been implicated. Possible mechanisms for this have been proposed including displacement of physical activity, reduction in resting energy expenditure compared to other activities, increasing sleep deprivation, exposure to advertising and consequent use of foods commonly advertised on television, and increased calorie intake while watching television [12] [13]. A number of studies have demonstrated small but significant positive associations between television viewing and body mass index (BMI) or body fatness of children and adolescents, particularly in adolescent females [13] [14] [15] [16]. Other studies have failed to demonstrate such an association in similar age groups [17] [18]. Meta-analyses have highlighted a similar variation of findings, but have concluded that on balance there is a relationship between television viewing and BMI. The public health importance of this relationship is suggested by our demonstration of an association between television viewing throughout childhood and adverse health indicators in adulthood, including obesity, decreased cardiovascular fitness, smoking, higher cholesterol levels and even poor educational achievement [19] [20] [21].

The International Study of Asthma and Allergies in Childhood (ISAAC) Phase 3 programme was originally designed to measure time trends in the prevalence and severity of asthma, rhinoconjunctivitis and eczema and to explore the relationship between lifestyle, other putative risk factors and the development of asthma and allergies [22]. It also provided the opportunity to explore the relationship between lifestyle factors such as time spent watching television and BMI, as data on both these measures were collected. Here we present analyses in both adolescents (aged between 12 and 15 years) and children (aged between 5 and 8 years). Our hypothesis was that longer periods of time viewing television would be positively associated with BMI in both age groups, and that this association would be observed worldwide. We also wanted to test whether females in both age groups had a stronger association between television viewing and BMI than males as had been observed in some previous studies [13] [23].

## Methods

ISAAC is a multicentre, multi-country, multiphase, cross-sectional study investigating the prevalence of the symptoms of asthma, rhinoconjunctivitis and eczema and the role of risk factors, and has previously been described [24]. ISAAC Phase Three used the Phase One standardised core questionnaire on symptoms of asthma, rhinoconjunctivitis and eczema, and included an optional environmental questionnaire (EQ) to collect potential risk factor specific etiological data including height, weight, and television viewing. The adolescents self-completed their questionnaires and parents or guardians completed questionnaires for the children. The questionnaires are on the ISAAC website isaac.auckland.ac.nz.

In this paper we focus on the reported time spent viewing television by the study participants and its possible relationship to their BMI. Television viewing was established through the following question: “During a normal week, how many hours a day (24 hours) do you (does your child) watch television?” The participants were asked to categorise their viewing time as “less than 1 hour”; “1 hour but less than 3 hours”; “3 hours but less than 5 hours”; or “5 hours or more”. These categories were designated as ‘short’, ‘moderate’, ‘long’ and ‘prolonged’ periods of television viewing.

The EQ included questions on height and weight which were self-reported by adolescents and reported by the parents for the children. In some centres, each subject's height and weight were measured objectively; there were no standardised or specific instructions for doing this.

Centres with at least 70% full data were included. Individuals without complete age, sex, television, height or weight data were excluded. To preserve as much remaining data as possible, but also to eliminate likely erroneous data, we applied the following thresholds:

- For adolescents in each centre, those in the top and bottom 0.5% of weights and heights, and those with heights less than 1.25 metres were excluded. BMI was calculated (weight (kg)/height (m)^2^), and BMIs less than 10 kg/m^2^ and greater than 45 kg/m^2^ were removed. This resulted in the exclusion of a further 4,219 adolescents.
- For children in each centre, those in the top and bottom 0.5% of weights and heights, and those with heights less than 1.0 metre were excluded. BMI was calculated, and BMIs less than 9 kg/m^2^ and greater than 40 kg/m^2^ were removed. This resulted in the exclusion of a further 1,601children.

### Statistical Analysis

BMI was assessed separately for each age group using a general linear mixed model with centre as a random effect and age, sex, television watching and measurement type as fixed effects. After finding an interaction between sex and the duration of television watching in the older age group, analysis was undertaken separately for males and females. The BMI values reported are the modelled means for those who viewed television for less than one hour per day for all ages in the adolescent and children's groups respectively. The analysis was repeated using only the centres that recorded height and weight by measuring rather than self-report. Each individual was classified as obese, overweight or otherwise [25] and the percentage in the three categories summarised by country. Then a general linearised mixed model with a log link and binomial distribution compared BMI in two categories – overweight and obese against normal or underweight, giving the relative risks and confidence intervals of the three higher television watching levels compared with the shorter television watching category.

## Results

### Participants

**Figure 1:**
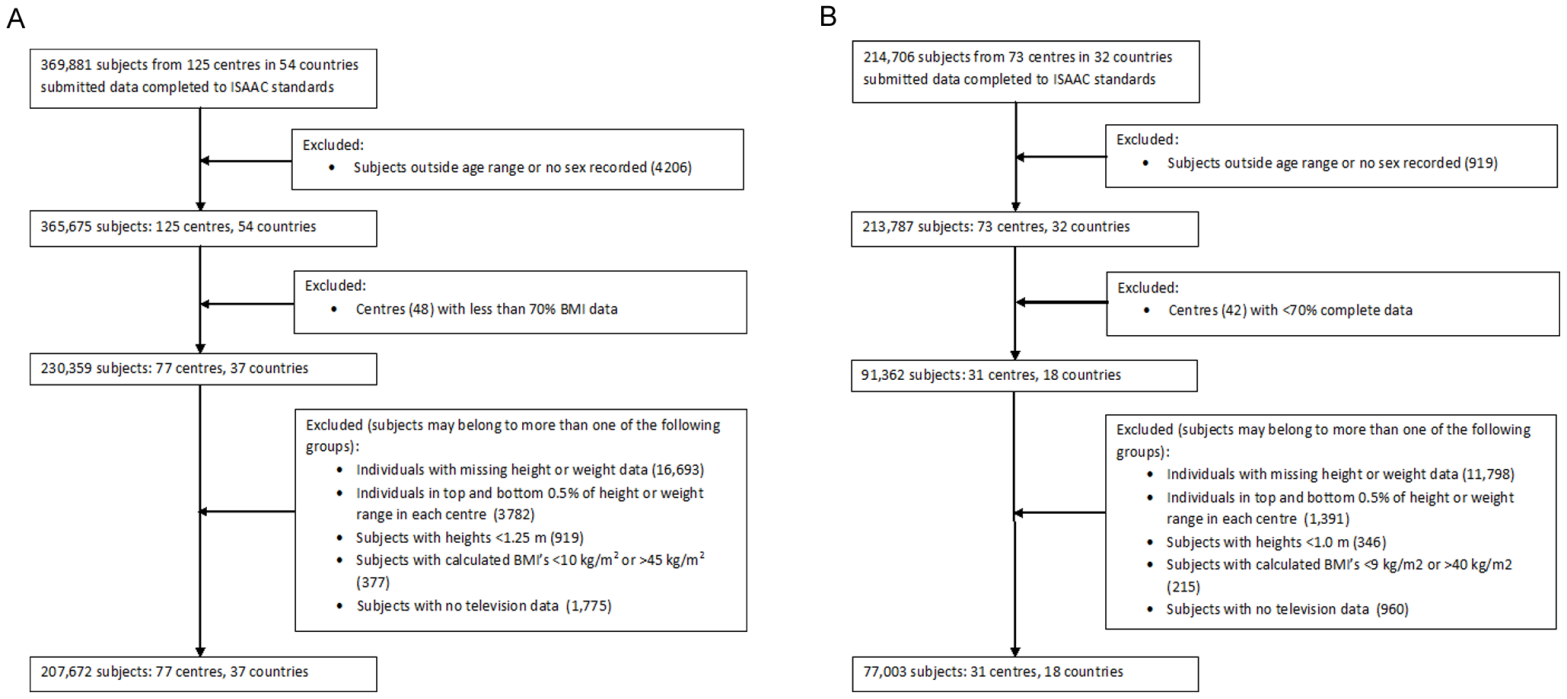
Flow of subjects through study. Panel A shows the flow of adolescents and Panel B shows the flow of children.

For the adolescents, data which included heights, weights and television watching were submitted from 125 centres in 54 countries (369,881 subjects). Following sequential application of the exclusion criteria, 207,672 adolescents (77 centres/37 countries) were included in the final analysis (Figure 1a). Of these 47,456 subjects from 17 centres reported measured height and weight data and 160,216 subjects from 60 centres provided self-reported height and weight data.

For the children, data which included heights, weights and television watching were submitted from 73 centres in 32 countries (214,706 subjects). Following sequential application of the exclusion criteria, 77,003 children (31 centres/18 countries) were included in the final analysis (Figure 1b). Of these 15,357 children from 7 centres reported measured height and weight data and 61,646 children from 24 centres provided parent-reported height and weight data.

### Television viewing

**Figure 2:**
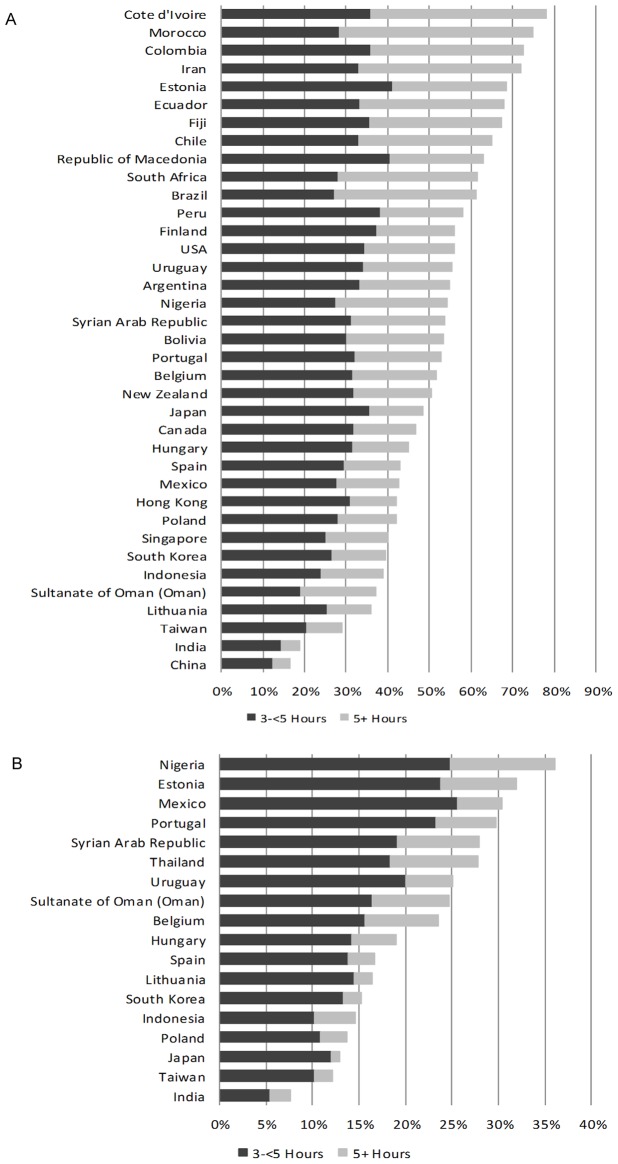
Daily television viewing of subjects by country presented as percent in long and prolonged categories. Panel A shows adolescent television viewing and Panel B shows television viewing of children.

In the adolescents, the mean percentage reporting moderate, long or prolonged viewing was 89%. The proportion of adolescents that reported long or prolonged television viewing ranged from 17% in China to 78% in Cote D'Ivoire. (Figure 2a).

In the children, the mean percentage reporting moderate, long or prolonged viewing was 79%. The proportion of children that reported long or prolonged television viewing ranged from 8% in India to 36% in Nigeria. (Figure 2b).

### BMI

**Figure 3:**
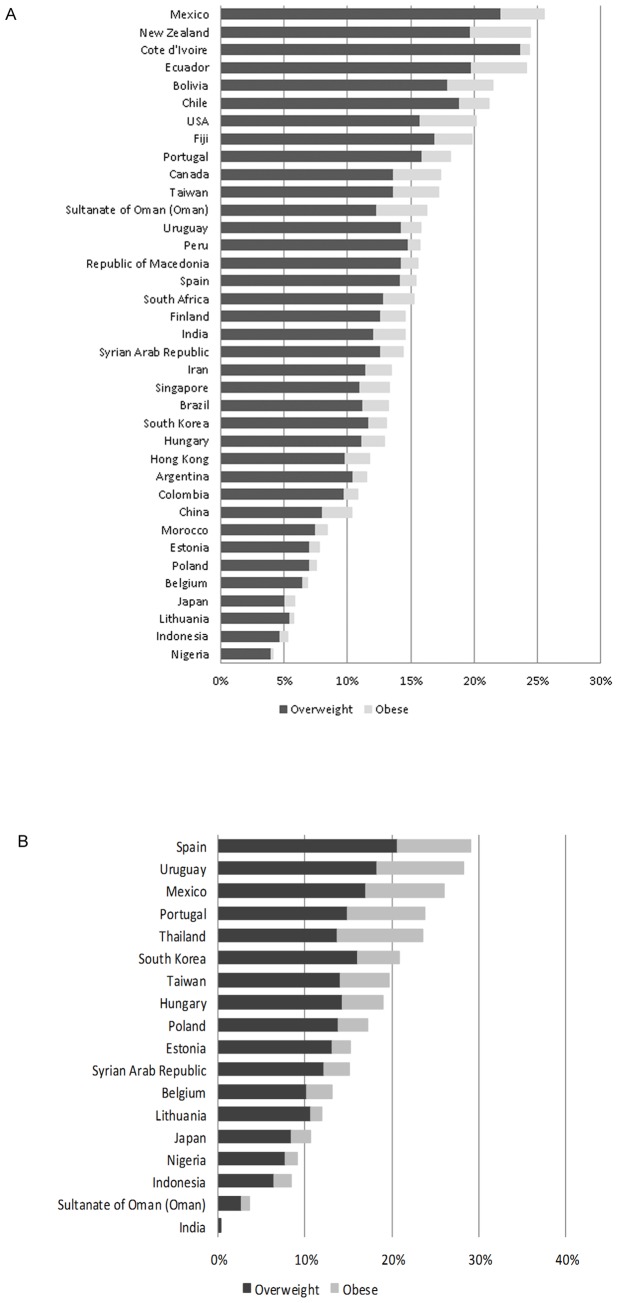
BMI of subjects by country presented as percent of subjects in overweight and obese categories. Panel A shows the BMI of adolescents and Panel B shows the BMI of children.

The proportion of adolescents who were overweight or obese ranged from 4% in Nigeria to 26% in Mexico. (Figure 3a) The proportion of children who were overweight or obese ranged from 0.3% in India to 29% in Spain. (Figure 3b).

### Television viewing and BMI – Adolescents

**Figure 4:**
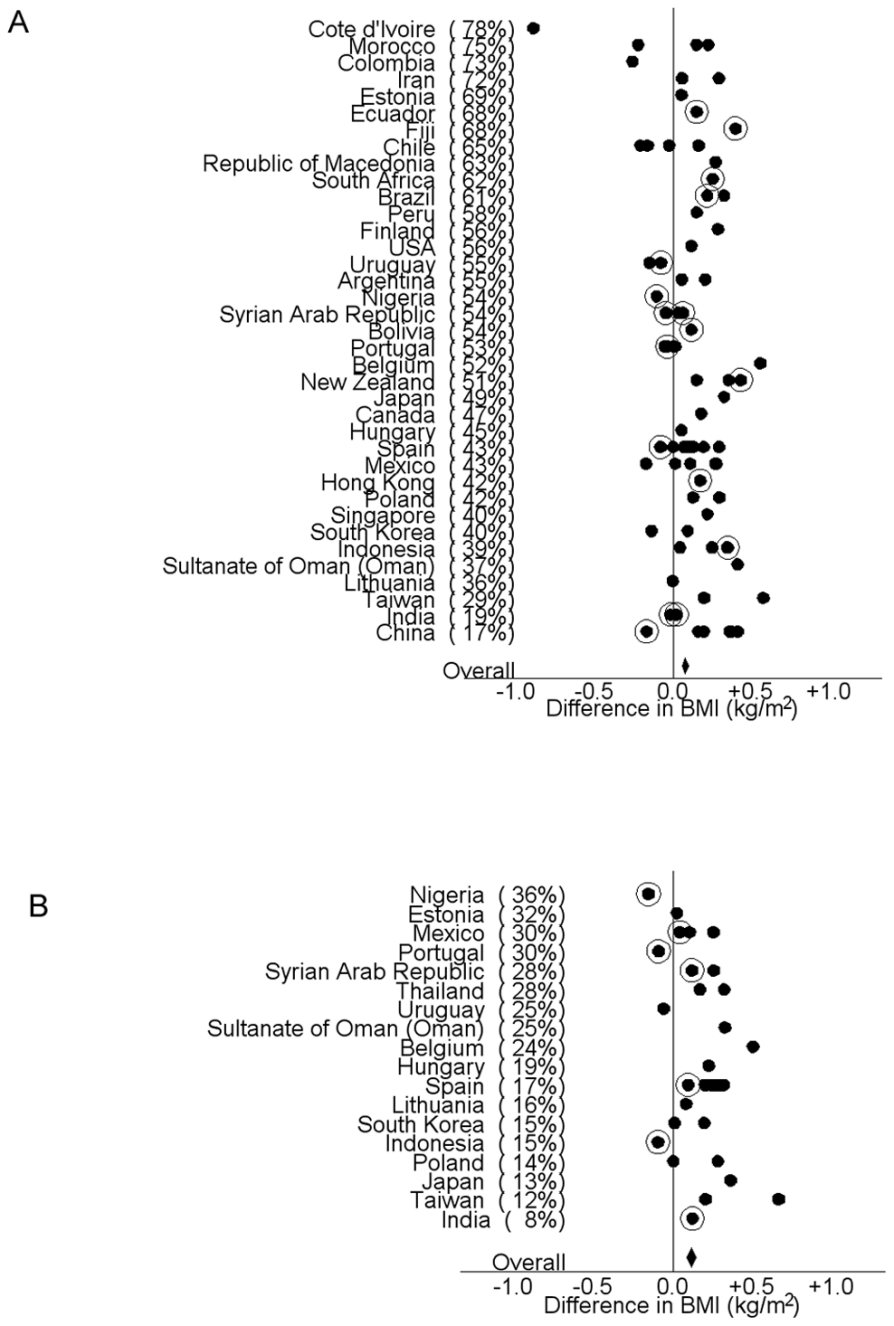
Association between average daily television viewing hours and subject BMI by centre. The difference in BMI (Kg/m^2^) between subjects with long and prolonged viewing hours and those with short and moderate viewing hours in each country by centre (positive difference represents an association of higher viewing time with higher BMI). Panel A shows the association in adolescents and Panel B shows the association in children. For each country the proportion of subjects who view television for more than three hours per day on average (long and prolonged categories combined) is shown in parentheses. Those centres with reported height and weights are shown with filled in circles, and those centres that measured heights and weights are shown with hollowed circles.

Figure 4a shows the difference in BMI (Kg/m^2^) between adolescents in the short or moderate viewing categories combined and those with long or prolonged daily television viewing, in each country by centre. There was a significant sex interaction between television viewing and BMI in the adolescent age group, (P<0.0001).

#### Females

Data were available for 104,712 adolescent females. The mean BMIs in the short viewing category were 18.9, 19.1, 19.6 and 20.0 kg/m^2^ for ages 12, 13, 14 and 15 respectively. After controlling for centre, age, and measurement type, there was a positive association between higher levels of television watching and BMI (p<0.0001) with an apparent dose response effect. (Table 1).

**Table 1:** Estimated BMI (SE) in kg/m^2^ for short television viewing and the increase in BMI (SE) for each television viewing category by age and sex.

	BMI kg/m^2^ (SE)	Change in BMI Kg/m^2^ (SE) compared with short viewing category		
	Short: (<1 hour/day)	Medium: (1–<3 hours/day)	Long: (3–<5 hours/day)	Prolonged: (5+ hours/day)
**Adolescents: Female**	19.97 (0.14)	0.14 (0.03)	0.21 (0.03)	0.30 (0.04)
**Adolescents: Male**	20.08 (0.16)	0.08 (0.03)	0.16 (0.03)	0.16 (0.04)
**Children: Female**	16.19 (0.18)	0.24 (0.03)	0.34 (0.04)	0.36 (0.06)
**Children: Male**	16.53 (0.18)	0.19 (0.03)	0.32 (0.04)	0.36 (0.06)

The BMIs stated in the adolescent short viewing category are for 13 year olds and in the children short viewing category are for 6 year olds.

#### Males

Data were available from 102,960 adolescent males. The mean BMIs in the short viewing category were 19.0, 19.2, 19.5 and 19.9 kg/m^2^ for ages 12, 13, 14 and 15 respectively. After controlling for centre, age, and measurement type, there was a positive association between higher levels of television watching and BMI (p<0.0001) with an apparent dose response effect. (Table 1).

### Television viewing and BMI – Children

Figure 4b shows the difference in BMI (Kg/m^2^) between children in the short or moderate viewing categories combined and those with long or prolonged daily television viewing, in each country by centre. There was no sex interaction found between television viewing and BMI in the children (p = 0.96). However, given that a sex interaction was found in the adolescent group, for consistency, we report our findings in the children by sex.

#### Females

Data were available for 38,272 female children. The mean BMIs in the short viewing category were 15.3, 15.3, 15.6 and 15.8 kg/m^2^ for ages 5, 6, 7 and 8 respectively. After controlling for centre, age, and measurement type, there was a significant positive association between higher levels of television watching and BMI (p<0.0001) with a dose response effect. (Table 1).

#### Males

Data were available for 38,731 male children. The mean BMIs in the short viewing category were 15.9, 15.6, 15.9 and 16.0 kg/m^2^ for ages 5, 6, 7 and 8 respectively. After controlling for centre, age and measurement type, there was a significant positive association between higher levels of television watching and BMI (p<0.0001) with an apparent dose response effect. (Table 1).

### Television viewing and BMI restricted to those with objective measures

25,667 adolescent females from 17 centres in 15 countries had their heights and weights measured rather than self-reported. This comprised 25% of all adolescent females. When analysis was restricted to this group, there remained a significant association between television viewing and BMI (p<0.0001).

21,789 adolescent males from 17 centres in 15 countries had their heights and weights measured rather than self-reported. This comprised 21% of all adolescent males. When analysis was restricted to this group, the association between television viewing and BMI was not significant (P = 0.40), but the estimated BMIs still showed an ordered effect.

7,770 female children (20%) and 7,587 male children (20%) from 7 centres in 7 countries had their heights and weights measured. When analysis was restricted to this group, there was no significant association between television watching and BMI for females or males (p = 0.25 and p = 0.95 respectively).

### Relative risks for overweight and obesity by age group and sex

Relative risks and confidence intervals by sex in each age group for overweight or obesity in each television viewing category relative to the short television viewing category are shown in Table 2.

**Table 2:** Relative risk (95% CI) for overweight or obese by television viewing category in both age groups, children are reported both combined and by sex, adolescents by sex only.

	Television Viewing Category			
Relative Risk of Overweight /Obese	Short: (<1 hour/day)	Medium: (1–<3 hours/day)	Long: (3–<5 hours/day)	Prolonged: (5+ hours/day)
**Adolescents: Female**	1.0	1.17 (1.04, 1.32)	1.27 (1.08, 1.48)	1.45 (1.21, 1.74)
**Adolescents: Male**	1.0	1.10 (1.0, 1.20)	1.14 (1.01,1.29)	1.11 (0.95, 1.29)
**Children: All**	1.0	1.24 (1.10, 1.40)	1.37 (1.20, 1.57)	1.35 (1.16, 1.58)
**Children: Female**	1.0	1.22 (1.07, 1.39)	1.34 (1.16, 1.55)	1.35 (1.10, 1.65)
**Children: Male**	1.0	1.27 (1.11, 1.45)	1.40 (1.21, 1.62)	1.36 (1.19, 1.56)

## Discussion

In this study there was a positive association between longer periods of television viewing and BMI in both adolescents and children worldwide. There was a dose response effect in both age groups. There was a 10 to 27% increased risk of overweight or obesity in adolescents and children watching 1–3 hours of television per day, with adolescent females having a 45% increased risk when watching more than 5 hours of television per day. The strength of these associations are consistent with those found in previous studies [15].

The cross sectional nature of this study prevents determination of whether these associations are in some way causal or whether television viewing may be a marker of other lifestyle factors that may influence BMI, such as other dietary factors, socioeconomic status, physical activity or other sedentary activities. However, the associations are consistent with longitudinal studies that demonstrate a temporal sequence between television viewing and the development of overweight and also with intervention research that shows that reducing viewing time can slow the increase in BMI in adolescence [26].

The stronger association between television viewing and obesity in adolescent females has been noted in previous studies [23]. Adolescent females watched about the same amount of television as their male counterparts, so the increased effect is not due to females watching more television than males. We can only speculate at the reasons for a stronger association in adolescent females which could include biological differences in the nature and timing of puberty that may make females in this age group particularly vulnerable to increasing body weight in response to excessive sedentary activity. Behavioural differences could also explain the weaker association in males in that adolescent males are more physically active than females [27] and those who watch a lot of televised sport may also participate in sport, mitigating the effect of sedentary behaviour on BMI.

The overall results of this study reinforce the findings of previous meta-analyses, where there is a relationship between television viewing and body fatness/BMI, most particularly in adolescent females [13] [28]. The magnitude of the associations we found were consistent with previous studies [28]. The public health significance of the effect of television on childhood obesity is suggested by its risk of adult obesity and its complications [7] [8] [29].

This study has also shown that long periods of television viewing are common in children, increasing substantially in adolescents. For example, 21% of children watched 3 hours or more of television daily, increasing to 52% of adolescents. This is consistent with levels that have been reported in previous studies from affluent countries, but also highlights the generally high levels of television viewing in middle and low income nations.

We have demonstrated a wide variation in the prevalence of overweight and obesity in different regions of the world, with an average of 14% of adolescents and 17% of children overweight or obese. We recognise that the observed rates are dependent on the defined geographical area, predominantly urban, studied in each centre, and may not necessarily be representative of the country as a whole particularly where there are few centres with a smaller number of participants.

### Strengths and limitations

The major strengths of this study were its size and multicentre structure, where 207,672 adolescents from 77 centres in 37 countries and 77,003 children from 31 centres in 18 countries were surveyed. Many of the centres were from middle and low income countries from which previous data on the association between television viewing and BMI had not been reported.

There are a number of important limitations to the study including its cross sectional design which allows identification of associations, but not assessment of temporal sequence or causality. The assessment of television viewing and BMI was undertaken by questionnaire, which were completed by the adolescents themselves and by parents on behalf of the children. There is the possibility of misclassification error in both questions, but this is more likely to have reduced any effect towards the null hypothesis.

Another limitation is that of the recording of height and weight – self-reported or measured in the adolescent group and parent-reported or measured in children. In the centres where height and weight were measured there were no standardised instructions or devices for doing this, due to the nature of the international study. It is likely that weight may be inaccurately reported, but these inaccuracies are likely to have reduced any effect towards the null hypothesis and thus result in an underestimation of the effect seen.

It is possible that a social desirability bias will have led to an underreporting of both weight and television viewing time among some participants. This also may have reduced the magnitude of the effect of television watching on BMI that we have demonstrated. Our analytical approach was to maximise data available for analysis, but also to apply upper and/or lower thresholds to heights, weights and calculated BMI's for each centre in order to exclude erroneous data. We were reassured that restricting analysis to measured heights and weights only for adolescents still resulted in significant association between television viewing hours and BMI for female adolescents, while an ordered effect remained for adolescent males. There was no significant association between television viewing and BMI in the group of children that had their heights and weights measured; this may be in part due to the small data set available for analysis.

This study only collected data on television viewing times, not other sedentary or ‘screen time’ activities like video games or computing. These do not appear to be as strongly associated with BMI as television [13], but it is possible that these have an increasing influence on children's body weight as the time spent in these activities increases. Males tend to play more video games than females [13] [27] [30] and this provides another potential reason for sex-differences in the association between television viewing and BMI in adolescence: if males with low reported television use spend a great deal of their time in other sedentary activities, this could obscure the association between television and BMI.

## Conclusions

This study has demonstrated a positive association between longer periods of television viewing and BMI in both adolescents and children with an apparent dose response effect. The association is strongest in adolescent females. This study has also demonstrated high levels of television viewing amongst adolescents and children worldwide, with variable rates of overweight and obesity between countries in both age groups.
